# miRTrail - a comprehensive webserver for analyzing gene and miRNA patterns to enhance the understanding of regulatory mechanisms in diseases

**DOI:** 10.1186/1471-2105-13-36

**Published:** 2012-02-22

**Authors:** Cedric Laczny, Petra Leidinger, Jan Haas, Nicole Ludwig, Christina Backes, Andreas Gerasch, Michael Kaufmann, Britta Vogel, Hugo A Katus, Benjamin Meder, Cord Stähler, Eckart Meese, Hans-Peter Lenhof, Andreas Keller

**Affiliations:** 1 Center for Bioinformatics, Saarland University, Campus E2 1, 66041 Saarbrücken, Germany; 2 Department of Human Genetics, Saarland University, 66421 Homburg/Saar, Germany; 3 Department of Computer Sciences, University of Tuebingen, Sand 13, 72076 Tübingen, Germany; 4 Department of Internal Medicine III, University of Heidelberg, Im Neuenheimer Feld 350, 69120 Heidelberg, Germany; 5 Siemens Healthcare, Hartmannstr. 16, 91052 Erlangen, Germany

## Abstract

**Background:**

Expression profiling provides new insights into regulatory and metabolic processes and in particular into pathogenic mechanisms associated with diseases. Besides genes, non-coding transcripts as microRNAs (miRNAs) gained increasing relevance in the last decade. To understand the regulatory processes of miRNAs on genes, integrative computer-aided approaches are essential, especially in the light of complex human diseases as cancer.

**Results:**

Here, we present miRTrail, an integrative tool that allows for performing comprehensive analyses of interactions of genes and miRNAs based on expression profiles. The integrated analysis of mRNA and miRNA data should generate more robust and reliable results on deregulated pathogenic processes and may also offer novel insights into the regulatory interactions between miRNAs and genes. Our web-server excels in carrying out gene sets analysis, analysis of miRNA sets as well as the combination of both in a systems biology approach. To this end, miRTrail integrates information on 20.000 genes, almost 1.000 miRNAs, and roughly 280.000 putative interactions, for Homo sapiens and accordingly for Mus musculus and Danio rerio. The well-established, classical Chi-squared test is one of the central techniques of our tool for the joint consideration of miRNAs and their targets. For interactively visualizing obtained results, it relies on the network analyzers and viewers BiNA or Cytoscape-web, also enabling direct access to relevant literature. We demonstrated the potential of miRTrail by applying our tool to mRNA and miRNA data of malignant melanoma. MiRTrail identified several deregulated miRNAs that target deregulated mRNAs including miRNAs hsa-miR-23b and hsa-miR-223, which target the highest numbers of deregulated mRNAs and regulate the pathway "basal cell carcinoma". In addition, both miRNAs target genes like PTCH1 and RASA1 that are involved in many oncogenic processes.

**Conclusions:**

The application on melanoma samples demonstrates that the miRTrail platform may open avenues for investigating the regulatory interactions between genes and miRNAs for a wide range of human diseases. Moreover, miRTrail cannot only be applied to microarray based expression profiles, but also to NGS-based transcriptomic data. The program is freely available as web-server at mirtrail.bioinf.uni-sb.de.

## Background

Gene expression profiles have gained increasing relevance over the last three decades and have become essential in modern biomedical sciences. About two decades ago, a further class of RNAs has been discovered: these non-coding oligonucleotides are indeed transcribed from the human genome, but no proteins are assembled according to their blueprints. MicroRNAs are a subgroup of these non-coding RNAs, currently attracting more and more attention. They have first been reported in a work by Ruvkun [1] and their first appearance in experiments has been associated with Lee et al [2].

MicroRNAs usually consist of 17 to 23 nucleotides and are detectable in the majority of human tissues and almost all bodily fluids [3-5]. It is known today that microRNAs influence the expression of target genes by binding to the corresponding mRNA, leading to its inactivation. Over 50% of all human coding genes seem to be targets of these short non-coding RNAs. MicroRNAs hereby help to control and fine-tune physiological cellular processes like differentiation, proliferation, or apoptosis. Nowadays, it also became apparent that microRNAs have a strong impact on pathological processes as well: Various microRNAs show altered expression patterns in human disorders including malignant [6-10], neurological [11], cardiovascular [12,13], or rheumatic diseases [14,15]. In order to get new insights into the molecular mechanisms leading to a specific disease, increasing attention is paid to the interaction of microRNAs and mRNAs of target genes.

**Figure 1:**
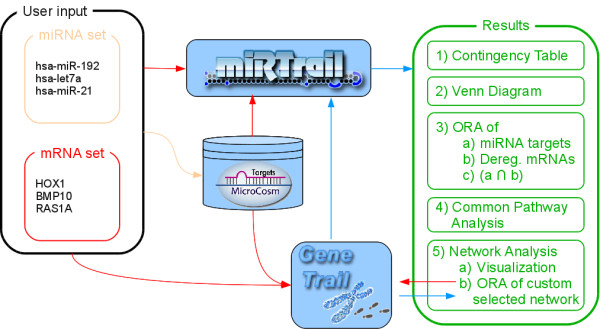
**Workflow**. Workflow of miRTrail. User submits two RNA sets (one is the set of deregulated miRNAs, the other is the set of deregulated mRNAs, both for the same disease). Orange color represents information flow of miRNA-related information: For each provided miRNA, the target mRNAs are determined (based on microCosm predictions or custom, uploaded interactions). This information then is used by miRTrail, indicated by the red arrow. In general, red color represents flow of mRNA-related information: The uploaded mRNA set as well as the miRNA targets are used in GeneTrail to perform ORAs as described in the "Methods"-section. Blue color represents information flow of results-related information, e.g. for the overlap of pathway sets. Finally in the results, the network analysis allows for targeted inspection of the provided information, based e.g. on miRNA families-related subnetworks. The modular design of miRTrail becomes visible here, also allowing for convenient extension of future analyses and usage for a diversity of different organisms.

The technologies that are most commonly applied to measure miRNA expression profiles are closely related to the methods for measuring gene expression profiles, namely quantitative real-time polymerase chain reaction (qRT-PCR) [16,17], oligonucleotide microarrays [18,19], and high-throughput sequencing [20,21]. These three technologies allow measuring the expression of sets of miRNA very efficiently. While qRT-PCR is mostly applied to rather small subsets of miRNAs, microarrays enable to profile the whole human miRNome and high-throughput sequencing is additionally applied to detect novel mature forms of miRNAs. Remarkably, with the still growing number of miRNAs, and the likewise growing number of biological experiments carried out with the above-mentioned high-throughput methods, and the manifold of possible interactions between miRNAs and mRNAs, computer aided analyses are essential to grasp the information hidden in the large data sets. Therefore, much ongoing work focuses on the combined analysis of miRNAs and their targets. Two classes of bioinformatics approaches related to this topic are 1) tools that aim at discovering the targets of miRNAs and 2) tools that aim at an integrative analysis of miRNA and mRNA sets. Algorithms belonging to the first class usually rely on sequence-complementarity and often also include thermodynamical aspects [22], machine learning [23-25], or experimental validation steps [26]. An overview of respective programs, including a comparison, can be found in [27]. Additionally, approaches primarily based on experiments are becoming prominent in recent years [28,29]. Naturally, these approaches are more likely to reveal significant miRNA - mRNA interaction pairs than computational approaches. However, they usually require not unimportant amounts of time and resources and, e.g. by design, might also miss relevant interactions. While not strictly being a tool for the discovery of targets or for an integrated analysis of miRNA and mRNA sets, TAM [30] offers enrichment analyses on miRNA sets, thus potentially paving the way to link common functions with related miRNAs. Tools for the second purpose, an integrative analysis of genes and their miRNA regulators, include MMIA [31], DIANA-mirExTra [32], or miRGator [33]. MMIA, allows to combine expression profiles of miRNA and mRNA experiments and then performs a pathway analysis on the intersection of the predicted target mRNAs and the according inversely correlated mRNAs. Additional analyses include Transcription Factor Binding Sites enrichment and diseases that are found to be associated with the inversely deregulated miRNAs. DIANA-mirExTra web-server integrates the potentially novel prediction of miRNAs having one or more of the submitted genes as their targets. This, likewise, allows shedding light on the function of the miRNAs. In detail, the algorithm investigates the 3' UTR sequences of deregulated genes and searches for over-represented six nucleotide long motifs, thus, enabling the identification of matching miRNAs. Finally, miRGator uses public expression data to analyze expression correlation between miRNA and target mRNA/proteins. The miRNA - target interactions are based on miRanda [22], PicTar [34], and TargetScanS [35] and the function of miRNAs is inferred from the related target mRNAs. To this end, a statistical enrichment analysis is performed for the established GO-terms, pathways, and also disease associations. Moreover, it integrates a first approach towards a manual inspection of the underlying network, offering vertex- or edge-filtering but, to-date, no ways to further cope with this information. Here, we present miRTrail (freely available to non-commercial users at mirtrail.bioinf.uni-sb.de), a knowledge-based tool for integrative network analysis that allows for studying the interactions between microRNAs and their target genes, and especially in the case of diseases, the implications of expression changes on pathogenic processes. Our tool excels by its broad functionality, as (1) it can be applied to a single disease or a group of diseases, (2) it covers a wide variety of biochemical categories, and it can be used to evaluate (3) qRT-PCR, microarray, as well as NGS-based transcriptome data. In its current stage, the organisms of Homo sapiens, Mus musculus, and Danio rerio are supported and further extension is continuing. While many solutions exist that provide either analyses of miRNAs, or mRNAs, or a combination of both, miRTrail allows for the simultaneous, combined statistical analysis of all of these three. A schematic description of its workflow is presented in Figure 1, depicting the integration of the provided data about miRNA and mRNA deregulation and the offered statistical analyses intended to facilitate the work with such complex information, especially when used in combination. As such, our tool is able to not only give initial but also thorough insights, even for a very detailed inspection of the given input based on the network analysis.

One of the original goals of our research was to improve the understanding on the molecular level of melanoma. Thus, as a first application, we investigated miRNA and mRNA expression profiles of this cancer entity, integrating information from 1) the gene expression omnibus GEO [36], 2) the PhenomiR 2.0 human miRNA and diseases database [37], 3) target prediction algorithms [38], and 4) biochemical pathway information of different resources integrated via the BNDB and GeneTrail [39]. By applying miRTrail to these data, we found highly significant coherences between dysregulated miRNAs and matching dysregulated targets of these miRNAs. An additional network analysis highlighted the potential implications of eight miRNAs via their target mRNAs on pathogenic processes in melanomas.

## Implementation

In this section, we start by describing the general idea behind miRTrail, followed by the data, techniques and tools that are used to provide the rich functionality, as well as information on the exemplary input data. Here, the input is originating from publicly available services like NCBI GEO and PhenomiR. Our tool is not restricted to these services, as they are intended for demonstration purposes. Especially, all of miRTrail's functionality is available for the organisms of Homo sapiens, Mus musculus, and Danio rerio, and can easily be extended to support other organisms in the future.

### Methodoloy - Multipartite graph

Our webservice miRTrail allows for the joint/integrated analysis of miRNA and mRNA entities, in respect to given diseases - since the latter protein-coding RNAs are targets of the former non-coding RNAs. We decided to realize the integration of data by constructing a graph or network. To be exact, an *r*-partite or multipartite graph *G*:

$$G=\left(V,E\right)$$

with:

• *V *= *V*_miR _∪ *V*_mR _∪ *V*_di_

• *V_X _*∩ *V_Y _*= ∅ for *X ≠ Y *and *X*, *Y *∈ {*miR, mR, di*}

• *E *= *E*_mR-di _∪ *E*_miR-di _∪ *E*_miR-mR_

• *E_X − Y _*= {(*u, v*)| *u *∈ *V_X _, v *∈ *V_Y _, X ≠ Y*} for *X, Y *∈ {*miR, mR, di*}

where the vertex-set *V *is the union of disjoint vertex-subsets, the edge-set *E *is the union of disjoint edge-subsets, and an edge only connects vertices of different vertex-subsets.

For each user and the according uploads, individual networks are created. An efficient and open-source interface for the creation of graphs is offered by the C++ Boost Graph Library and the associated adjacency_list-construct.

### MicroRNA - Target mRNA Interactions

The actually known 20,000 genes and 1,000 miRNAs allow for 20 million possible interaction pairs, where a miRNA may regulate a gene. To find the most reliable candidates, prediction algorithms have been developed. One of the most prominent algorithms for miRNA - target mRNA interactions is the miRanda algorithm and the respective web-resource microCosm [38]. The miRanda algorithm is sequence-complementarity based and includes a thermodynamic analysis of the miRNA - target mRNA complex. The results are then post-processed by a filtering on conservation of the target site. MicroCosm offers miRNA - target mRNA interactions in combination with a p-value threshold. In the beginning, we decided to perform analyses for three thresholds (0.01, 0.001, 0.0001), and extracted all interactions having a value smaller than the respective alpha level, yielding 279,225, 85,050, and 26,984 interactions, respectively. Because of the heterogenity of the expression data, we finally chose to use a threshold of 0.01, in turn leading to approximately 400 target mRNAs per miRNA in human. The appropriate predictions for the other supported organisms are automatically selected by miRTrail according to the organism in the identifiers of the uploaded miRNA deregulation information.

Alternatively, custom pairwise miRNA - target mRNA interactions can be uploaded in a tab-delimited format, thus allowing e.g. for the use of experimentally validated interactions. Details on the exact format for this input can be found on the homepage of miRTrail, especially regarding gene and miRNA identifiers.

### Analysis of independance

Based on the pairwise miRNA - target mRNA interactions (for a custom prediction-threshold (default of 0.01) or from a custom list provided as upload), the miRNA and (target) mRNA of each pair is compared to the input in order to see if it is up-, down- or not deregulated. This information is tabulated in a contingency table to provide an overview to the user. An according p-value is calculated, based on a Chisq-distribution with 4 (6 - 2) degrees of freedom. Given that the uploaded information about dysregulated genes/mRNAs only contains entries with the same direction of deregulation, the computation of an according p-value is not allowed by miRTrail and no p-value will be displayed, but instead a note for the user. However, the table will nevertheless be displayed as an overview.

To help the researcher get an impression about the influence of the used miRNA - target mRNA interactions in this step, especially when using data from prediction algorithms, we offer an option to randomize upon the provided data of miRNA and mRNA deregulation. The deregulation pattern (genes/miRs being up- or downregulated) is kept as-is while the identifiers are sampled at random. This functionality is available via the "Randomize"-button.

### MicroRNAs from PhenomiR

For integrating dysregulated miRNAs, we used PhenomiR 2.0 (last update: 2011-02-15). This service offers manually curated data about differential regulation for a variety of diseases.

Specifically, we used the data for entry/ID: 639, concluding the results of a published melanoma study based on microRNA low density arrays, including 666 microRNAs. Selection of the statistically significantly dysregulated miRNAs in the miRNA extracts of adult melanoma patients and benign nevi controls was done with univariate Two-sample T-test and a significance level of 0.05. The size of the patient samples is 10 and 4 for the control, respectively. An overview of the numbers of up- and downregulated miRNAs can be found in Table 1.

**Table 1:** Summary of deregulated genes and miRNAs

	# up-reg	# down-reg	sum of up and down
**genes**	2550	2218	4768

**miRNAs**	16	17	33

Deregulated genes (from NCBI GEO) (α = 0.05) and deregulated miRNAs (Phenomir 2.0).

### MessengerRNAs from NCBI's GEO

The NCBI Gene Expression Omnibus (GEO) is a public repository providing data from microarray experiments, next-generation sequencing, and other high-throughput functional genomic data. The microarray experiments, in particular, must comply with MIAME guidelines in order to be accepted by NCBI's GEO.

We extracted the microarray expression profiles from data set GDS1375 (series published: 2005-08-25), including 63 arrays for 45 melanoma and 18 benign nevi samples. Due to possible variations between the experiments, we carried out a quantile-normalization of the expression values for all genes present on the respective data set. Selection of differentially expressed genes was performed on the normalized data using the univariate Two-sample T-test and a significance level of 0.05. An overview of the numbers of up- and downregulated genes can be found in Table 1.

### GeneTrail

The gene set analysis tool GeneTrail has been developed to help in the analysis of readily available or newly created high-throughput data. It allows for a comprehensive and efficient statistical evaluation of large genomic or proteomic datasets and covers a plethora of biological categories and pathways, e.g. KEGG, TRANSPATH, TRANSFAC, and GO. Analyses can be either performed via an 'Over-Representation Analysis' (ORA) comparing a reference set of genes to a test set or a 'Gene Set Enrichment Analysis' (GSEA) based on a sorted list of genes. While the calculation of ORA p-values relies on Hypergeometric distribution, many existing tools offer the calculation of GSEA p-values based on permutation tests, usually limited to a fixed number of permutations for performance reasons. GeneTrail integrates an exact calculation [40] corresponding to a commonly used non-parametric unweighted permutation test. This calculation is based on dynamic programming and thus allows, especially for large sets, a higher accuracy than by using a fixed number of permutations.

Recently, GeneTrail has been extended to directly allow the analysis of expression data originating from the NCBI GEO, resulting in GeneTrail Express [41]. This integration greatly facilitates the selection of differentially regulated genes and allows for a fast evaluation of the expression profiles in respect to biological categories and pathways.

### Visualization: BiNA and Cytoscape-web

While computational approaches are very important in contemporary research, manual inspection is often desireable to support the automatic analyses or to identify new aspects. To this end, we decided to include the visualization of the resulting interaction network of miRNAs and their (putative) targets. Due to the large amount of integrated data, efficient means for focusing are crucial. Therefore, we provide respective subnetworks, depending either on the choice of individual miRNAs or on members of miRNA-families contained in the input. Furthermore, only deregulated miRNAs are respected that are connected to deregulated target mRNAs, either by the prediction algorithm or the provided custom interactions, as we envision these entities and relations as the most relevant. For the actual visualisation, two selections are available for the user: BiNA and Cytoscape-web.

BiNA is a visualization and analysis tool for various biological networks. We developed a plug-in for the Java Webstart version of BiNA, which takes the miRTrail results and uses the visualization capabilities of BiNA for presenting the network. The user can choose between different graph layouts (organic, hierarchic, and orthogonal) and can modify the visualization in many ways. By default, the target-mRNA nodes are sized according to their degree for easier retrieval of high-degree nodes. It is also possible to save the network in different file formats for reusing the data in other tools or BiNA again. For larger graphs, this visualization-option is probably beneficial.

Cytoscape-web is modeled after the Cytoscape Java network visualization and analysis software [42]. Its JavaScript API allows for an integration into HTML-pages and convenient display of networks. We offer the user a choice of three different graph layouts (Circular, Radial, Tree) and the possibility to select the first neighbors of a selected node. Zoom and pan functionality is available and target-nodes are also sized according to their degree. As the graph is directly displayed in the browser-window, this visualization is especially suitable for a quick inspection of the network. Finally, we implemented context-menu items that greatly facilitate the search for related publications by performing NCBI PubMed queries ("inclusive" or "exclusive") for a custom selection of miRNA and mRNA nodes, given a disease was specified in the input.

## Results and Discussion

In the following, we will describe the range of different functions offered by miRTrail. Subsequently, an analysis of cutaneous malignant melanoma versus benign nevi is performed to illustrate the potential of our tool.

### Functionality of miRTrail

The miRTrail webserver recieves two dysregulation sets in separate text-files as input, one is the set of dysregulated miRNAs and the other is the set of dysregulated genes. For each uploaded identifier (for miRNAs, the standard annotation of miRBase is used, for genes the HGNC GeneSymbol annotation, respectively), the information whether the respective gene/miRNA is upregulated ('1') or down-regulated ('−1') has to be provided in the files by the user. Optionally, the disease of interest can be specified to allow for convenient NCBI PubMed queries for related information.

As the next step, the user can either choose a target-prediction threshold for microCosm targets predictions or can provide a list of custom pairwise miRNA - target mRNA interactions, potentially originating from proprietary experiments or other prediction algorithms. The default threshold for microCosm targets predictions is 0.01, amounting to around 280,000 miRNA - target mRNA interactions. Here, the user can also opt-in for a thorough GeneTrail analysis. Based on this information, the analyses are then carried out and, finally, the user is directed to the results. These will be stored uniquely for each analysis performed and can be shared with others by simply providing them with the link of the results page. The results presented herein can be reproduced using the example files provided by miRTrail.

The first provided analysis computes a contingency table relating the dysregulation of miRNAs and the dysregulation of target mRNAs and calculates the according p-value, based on a χ^2 ^distribution. This analysis allows for estimating whether there is an independance in the deregulation of the miRNAs and the target mRNAs.

Second, a Venn diagram is computed, providing the dysregulated genes that are targets of dysregulated miRNAs (overlap of the diagram), the not-dysregulated targets of dysregulated miRNAs (left part of the diagram) and the dysregulated genes that are not targets of the dysregulated miRNAs (right part of the diagram). For this Venn diagram, a p-value using the Hypergeometric distribution is calculated to show whether there exists a significant overlap between dysregulated genes and targets of dysregulated miRNAs. Third, gene set enrichment analyses for three gene sets are carried out using the comprehensive functionality of GeneTrail. Independently of each other, a so-called Over-Representation Analysis (ORA) - based on the Hypergeometric distribution - is carried out for the dysregulated genes, targets of dysregulated miRNAs and dysregulated targets of dysregulated miRNAs. In all cases, the gene sets are tested for significant enrichments/depletions in KEGG pathways. If the user previously decided to perform all GeneTrail analyses, the results will also include information about GO terms, TransPath pathways, transcription factors from Transfac, SNPs, and chromosomal location, among many others. By clicking on the 'details' button, the complete list of results is provided. Moreover, an overview showing the biological categories being significant in at least two of the three analyses is created. The "code" represents in which of the pairwise overlaps the respective category was found, similar to the file-permission scheme in Linux. So, a code of "2" e.g. shows that a category was found in the enrichment analysis of the dysregulated targets of dysregulated miRNAs as well as in the results of the dysregulated genes. A code of "3" would hence mean that this category was additionally found in the results of the targets of dysregulated miRNAs. Accordingly in Table 2, e.g. the "DNA replication" pathway was found to be enriched for dysregulated targets of dysregulated miRNAs as well as for the dysregulated genes/mRNAs.

**Table 2:** Overlapping pathways

Pathway	Related mRNA set	Code
Olfactory transduction 04740	a, b, c	7

DNA replication 03030	b, c	2

Lysosome 04142	b, c	2

Prostate cancer 05215	b, c	2

Small cell lung cancer 05222	b, c	2

Systemic lupus erythematosus 05322	b, c	2

a: Pathways related to targets of dereg. miRNAs

b: Pathways related to dereg. targets of dereg. miRNAs

c: Pathways related to dereg. mRNAs

Code: Arithmetic sum of the following:

1: Found for a and b

2: Found for b and c

4: Found for a and c

**Figure 2:**
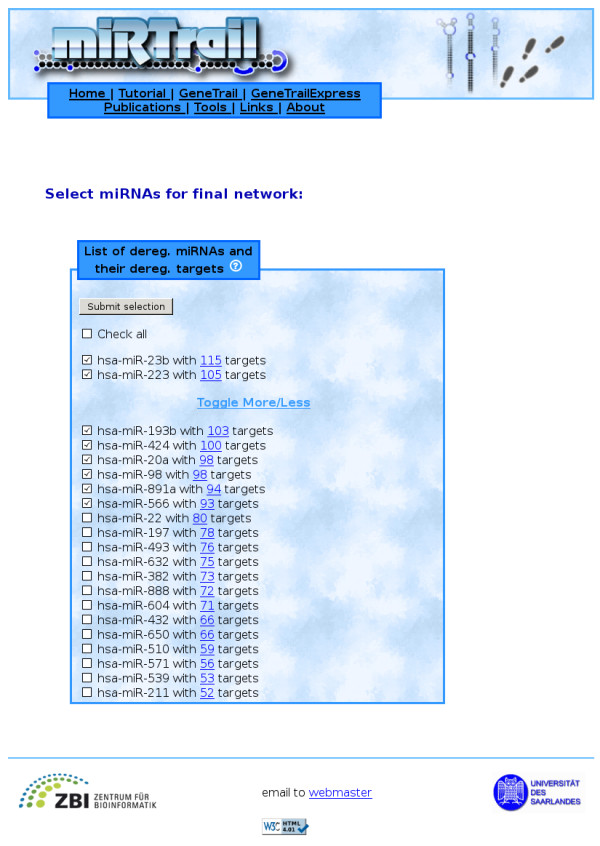
**MiRNA selection (individual)**. Demonstrates the selection of miRNAs of interest. Link next to the each miRNA shows the respective dysregulated target mRNAs.

**Figure 3:**
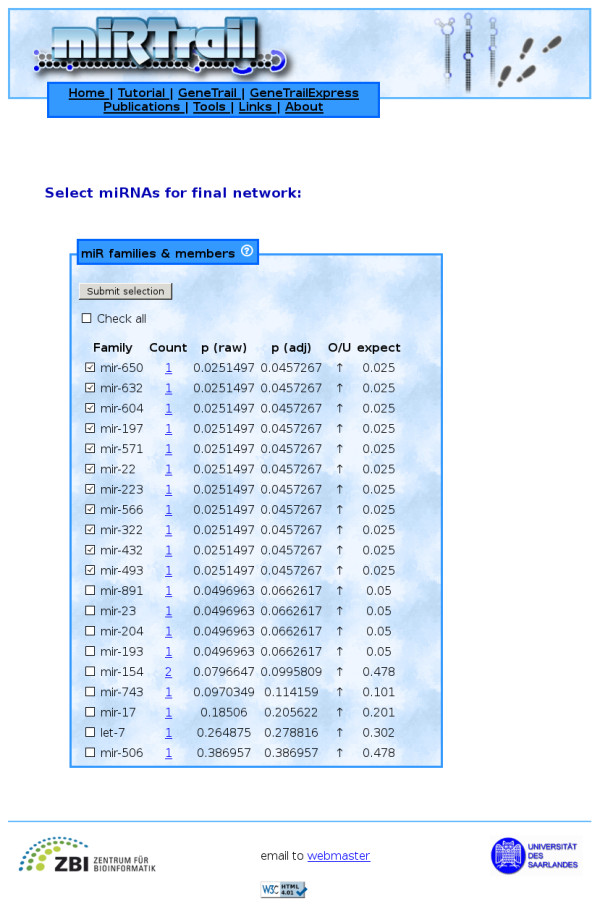
**MiRNA selection (families)**. Demonstrates the selection of miRNAs based on enriched miRNA families. All significant families (p(adj) *<*0.05) are preselected.

Finally, we carry out an integrative network-analysis approach on the comprehensive network containing dysregulated genes, dysregulated miRNAs, and the target interactions between them. A subset of interesting miRNAs and their according targets is selected as well as a custom degree constraint. The subset can be constructed either by selecting individual miRNAs, s. Figure 2, or miRNA families based on an Over-Representation Analysis of miRNA-family data from miRBase (miFam.dat) [43], s. Figure 3. The custom degree constraint allows the selection of the genes being the target of at least as many miRNAs as specified by the parameter. Based on this selection, using the Java Webstart-based viewer BiNA [44] or the web-based viewer Cytoscape-web [45], we show the resulting network, allowing for a manual inspection of the inherent interactions. In the network visualizations, nodes with rectangular shapes belong to miRNAs, nodes with round shapes to genes, red color means up-regulation, green color means down-regulation, and genes and miRNAs are connected by edges if a putative miRNA - target interaction exists. Additionally, a more fine-grained ORA is available, being performed only on the genes that are contained in the custom selection, which is also separately available as a list. These genes are assumed to be the most disease-relevant as they are found to be deregulated and simultaneously putative targets of deregulated miRNAs while, at the same time, being central to the network, according to their degree. This list is analysed for enrichments/depletions in KEGG pathways, Gene Ontology terms, OMIM disease relations, and NIA human disease gene sets. A thorough GeneTrail analysis can also be chosen here.

### Melanoma case study

We compared the expression profiles of cutaneous malignant melanoma to those of benign skin nevi samples from adult patients. While the proportion of melanoma cases among skin cancer patients is rather low (4%), it accounts for almost 75% of all skin cancer-related deaths. Even more, the prognosis for advanced melanoma is very poor (5-year survival-rate is only 5%) [46]. Hence, we decided to validate our tool based on melanoma data and to identify new aspects of this disease, potentially helping in the creation of promising new therapies for advanced melanoma patients.

**Figure 4:**
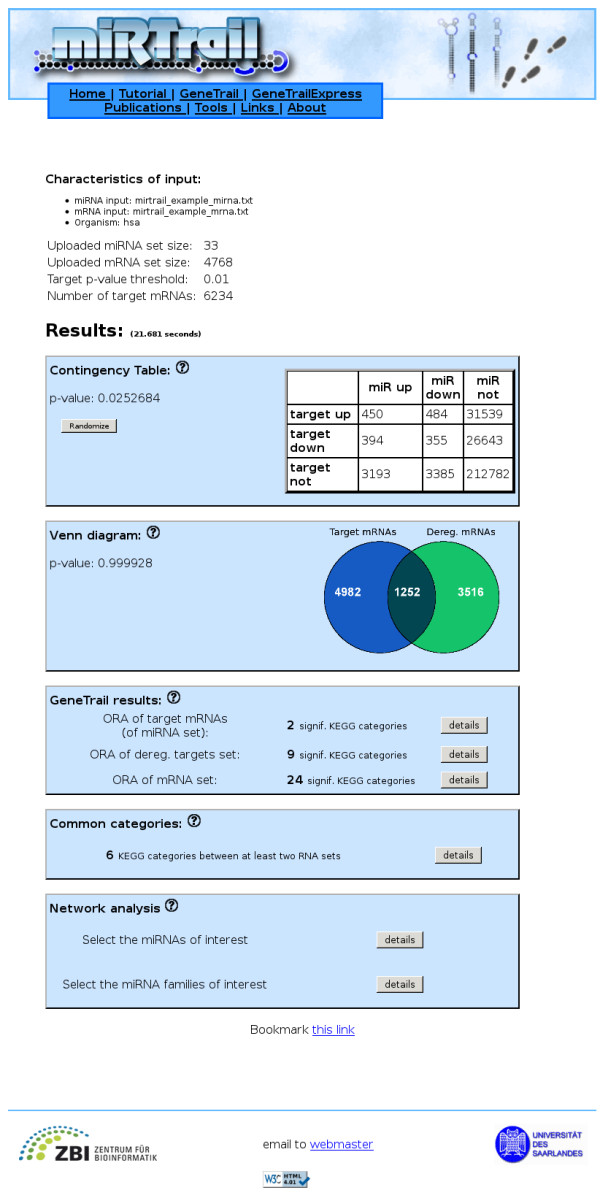
**Results Page**. This illustrates the results page of a miRTrail analysis. The network visualization and subsequent analyses are available in the subpanel at the bottom.

An illustration of the the results page for the miRTrail analysis on the melanoma miRNA and mRNA samples, as mentioned in the previous section, can be seen in Figure 4.

#### Analysis of independance

Using our tool, for the melanoma samples and a prediction threshold of 0.01 for the human miRNA - target mRNA interactions from MicroCosm targets, we were able to find statistical evidence about the dependance of deregulation of miRNAs and target mRNAs. The contingency table yielded a p-value of 0.025 (α = 0.05). Interestingly, independant of the miRNA being up- or downregulated, similar amounts of interactions were found for targets, then, being up (450 and 480), down (394 and 355), or not deregulated (3193 and 3385), respectively. In turn, more targets were found to be up-regulated for dysregulated miRNAs as well as for not dysregulated miRNAs. Surprisingly, 805 (450 + 355) interactions were found with both miRNA and predicted target, being deregulated in the same direction. Finally, as expected, a large number of interactions was found were both, the miRNA and the predicted target, were not deregulated (212782).

#### ORA of the three mRNA sets

Inspecting the ORA results, showed one KEGG pathway (GPI-anchor biosynthesis) to be significantly enriched for the set of targets of dysregulated miRNAs. In turn, the over-representation analysis of dysregulated targets of dysregulated miRNAs revealed nine significant pathways, including cancer related categories, like Non-small cell lung cancer, Prostate cancer, Small cell lung cancer, Endometrial cancer, and Glioma as well as enrichments in the Lysosome pathway and DNA replication. The analysis of the dysregulated mRNAs revealed the highest number of statistically signifi cant KEGG categories with a total of 24. Here again, several cancer-related pathways were found to be enriched as well as pathways like Cell cycle, Focal adhesion, or even signaling pathways (e.g. TGF-beta signaling pathway).

The result of the pairwise overlaps of the resulting pathway sets is described in Table 2. Among those, the DNA replication pathway [47] as well as the Lysosome pathway [48,49] have already been attributed to melanoma.

#### Network analysis

From the 33 input miRNAs, 21 were found to have dysregulated targets for a prediction threshold of 0.01. The miRNA with the least dysregulated targets was hsa-miR-211 (52 targets) while hsa-miR-23b was the miRNA with the most dysregulated targets (115). For this analysis, we decided to use the eight miRNAs having more than 80 dysregulated targets (miR-23b [50], miR-223, miR-193b [51], miR-424, miR-20a [52], miR-98, miR-891a, and miR-566), see Figure 2. We left the custom degree constraint at the default of 1 for the subsequent ORA. The resulting mRNA set comprises the dysregulated mRNAs that were predicted targets of at least one of the eight earlier miRNAs. Specifying a higher constraint would lead to a smaller network with only the mRNA nodes being targets of at least as many miRNAs as specified by this parameter and the according miRNA nodes, respectively.

##### ORA of subnetwork

The analysis of KEGG pathways showed significant enrichments for the three cancer-related categories: Prostate cancer, Non-small cell lung cancer, and Endometrial cancer. These categories were found to be enriched for genes that were deregulated while being targets of deregulated miRNAs, hence, the genes that are assumed to be the most disease-relevant due to their joint deregulation.

A total of 274 GO terms were found to be enriched or depleted for all of the three GO-trees, with enrichments in anti-apoptosis, cell proliferation, cell cycle, transcript initiation, RNA elongation, and regulation of translational initiation among others in the biological subtree.

Furthermore, an enrichment (RASA1 and PTCH1) for "Susceptibility to basal cell carcinoma" was found in the OMIM categories.

##### Visualization

**Figure 5:**
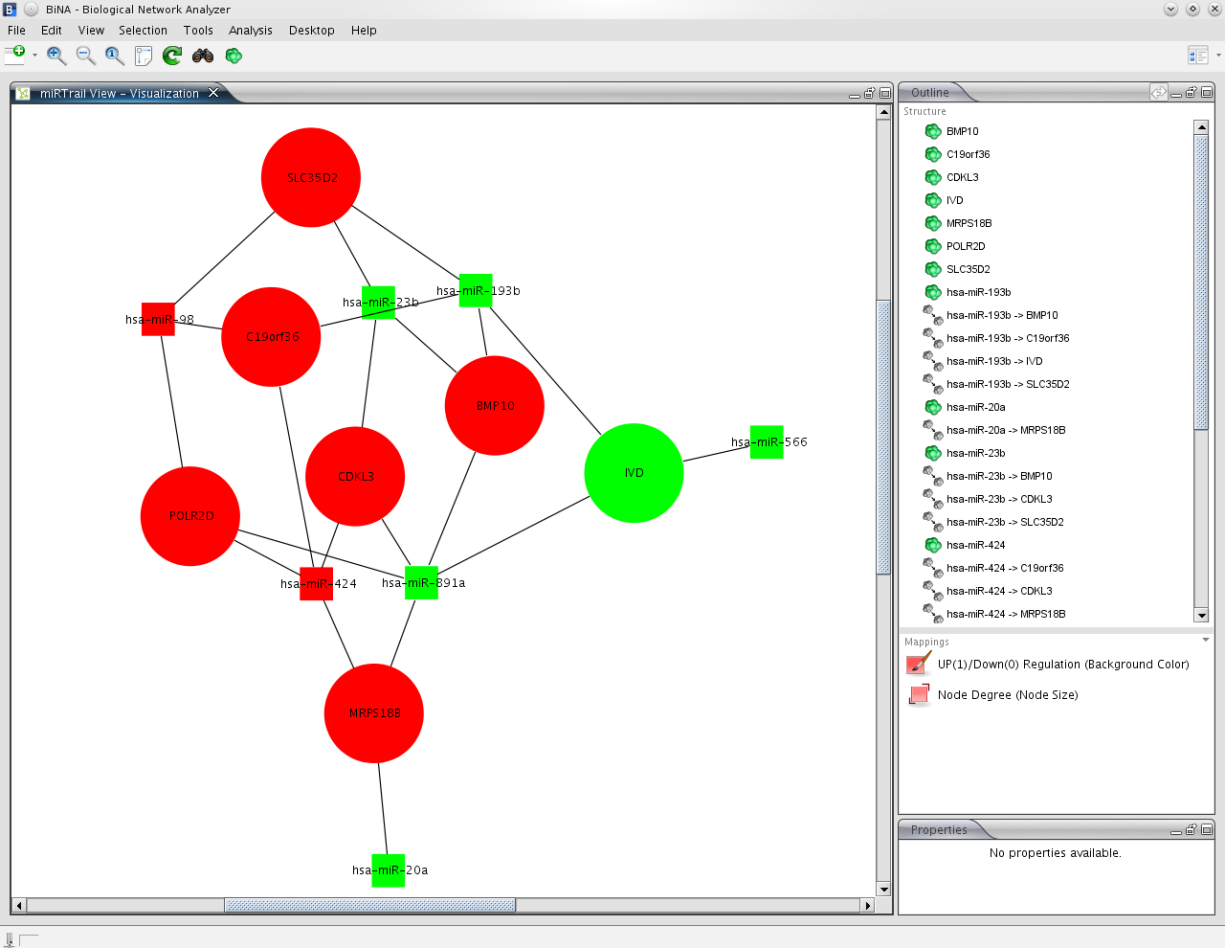
**Visualization: BiNA**. Subnetwork of top-8 miRNAs and a degree constraint for the target mRNAs of 3, thus, only seven miRNA nodes are displayed. Round shape represents mRNAs, rectangular miRNAs, respectively. Red color indicates upregulation, green color downregulation, respectively. Size of the mRNAs is according to their degree.

**Figure 6:**
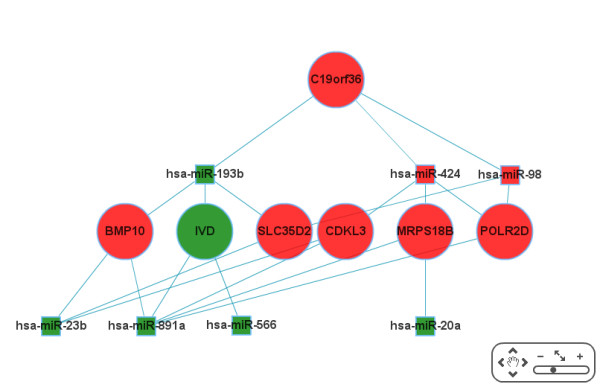
**Visualization: Cytoscape-web**. Subnetwork of top-8 miRNAs and a degree constraint for the target mRNAs of 3, thus, only seven miRNA nodes are displayed. Round shape represents mRNAs, rectangular miRNAs, respectively. Red color indicates upregulation, green color downregulation, respectively. Size of the mRNAs is according to their degree.

For this step, we decided to focus on smaller miRNA and mRNA sets to increase the visibility. However, also large selections can be efficiently handled and used for detailed manual inspections. Exemplary visualization can be found in Figure 5 and 6, for BiNA and for Cytoscape-web, respectively.

## Conclusions

The constantly increasing availability of data from different origins and of different nature allows for more complex and comprehensive analyses. To this end, we developed miRTrail to integrate information about RNA deregulation in diseases and putative interactions of miRNAs and mRNAs. Our tool provides a large collection of different analyses and performs in a transparent way, requiring only minor activity by the user, while offering versatile results. This greatly facilitates the adaption of this tool as it does not require complicated initial learning. Via the visualisation component, miRTrail enables the user to easily inspect the interactions and, thus, also to further process upon the selection.

MiRTrail - results will also be of great help in any scheme that aims in experimental confirmation of miRNA-targets. The final proof, here, requires extended experiments including the identification of the specific targeted region of a gene by in vitro binding and the analysis of in vivo effects by altered miRNA expression. The melanoma case study shows that we were able to detect highly significant results, despite the fact that we did not use autologous samples. This sets the ground for specific experimental assays that focus on significant miRNA - mRNA interactions in this tumor type. Hence, miRTrail is of great interest for the life sciences community as it can use data from next-generation sequencing, qRT-PCR, or microarray experiments.

## Availability and requirements

Project name: miRTrail

Project home page: http://mirtrail.bioinf.uni-sb.de

Operating system(s): Platform independent

Programming languages: C++, php

Other requirements: JavaWS version 1.6 or higher

## Authors' contributions

CL implemented the underlying framework as well as the web-interface. PL and NL performed the melanoma data analysis. AG and MK developed the BiNA visualization component. JH, BV, and BM tested and benchmarked miRTrail and took part in the writing of this manuscript. HAK collaborated in the project and the overall study and contributed to this manuscript. Support in the implementation of GeneTrail-related components came from CB. CS contributed the conceptual design of this study. EM, HPL, and AK developed the overall study and contributed to this manuscript. AK further supported the analysis and implementation of the herein presented solution. EM, HPL, and AK are the senior authors. All authors read and approved the final manuscript.
